# Characterization of COVID-19 in Assisted Living Facilities — 39 States, October 2020

**DOI:** 10.15585/mmwr.mm6946a3

**Published:** 2020-11-20

**Authors:** Sarah H. Yi, Isaac See, Alyssa G. Kent, Nicholas Vlachos, J. Carrie Whitworth, Kerui Xu, Katryna A. Gouin, Shirley Zhang, Kara Jacobs Slifka, Ann Goding Sauer, Preeta K. Kutty, Joseph F. Perz, Nimalie D. Stone, Matthew J. Stuckey

**Affiliations:** 1 CDC COVID-19 Response Team.

The coronavirus disease 2019 (COVID-19) pandemic has highlighted the vulnerability of residents and staff members in long-term care facilities (LTCFs) (*1*). Although skilled nursing facilities (SNFs) certified by the Centers for Medicare & Medicaid Services (CMS) have federal COVID-19 reporting requirements, national surveillance data are less readily available for other types of LTCFs, such as assisted living facilities (ALFs) and those providing similar residential care. However, many state and territorial health departments publicly report COVID-19 surveillance data across various types of LTCFs. These data were systematically retrieved from health department websites to characterize COVID-19 cases and deaths in ALF residents and staff members. Limited ALF COVID-19 data were available for 39 states, although reporting varied. By October 15, 2020, among 28,623 ALFs, 6,440 (22%) had at least one COVID-19 case among residents or staff members. Among the states with available data, the proportion of COVID-19 cases that were fatal was 21.2% for ALF residents, 0.3% for ALF staff members, and 2.5% overall for the general population of these states. To prevent the introduction and spread of SARS-CoV-2, the virus that causes COVID-19, in their facilities, ALFs should 1) identify a point of contact at the local health department; 2) educate residents, families, and staff members about COVID-19; 3) have a plan for visitor and staff member restrictions; 4) encourage social (physical) distancing and the use of masks, as appropriate; 5) implement recommended infection prevention and control practices and provide access to supplies; 6) rapidly identify and properly respond to suspected or confirmed COVID-19 cases in residents and staff members; and 7) conduct surveillance of COVID-19 cases and deaths, facility staffing, and supply information (*2*).

LTCFs comprise a broad range of nursing and residential care facilities that provide varying degrees of health and social services. LTCFs include ALFs and similar residential care facilities, SNFs and other nursing homes, and residential facilities for persons with intellectual and developmental disabilities. As of 2016, the 28,900 U.S. ALFs accounted for approximately 44% of the nation’s LTCFs and had 811,500 residents and 298,800 full-time equivalent care staff members (*3*). Resident care in ALFs is focused on activities of daily living, such as bathing and toileting, and assisting with skills needed for independent living, such as medication management and housekeeping (*3*). As of 2016, 52% of ALF residents were aged ≥85 years, 30% were aged 75–84 years, 71% were female, 81% were non-Hispanic White, and 17% had Medicaid as payer for services.

By November 6, 2020, approximately 569,000–616,000 COVID-19 cases and 91,500 deaths were reported among LTCF residents and staff members in the United States, accounting for 6% of total state COVID-19 cases and 39% of deaths (*4*,*5*). Although U.S. LTCF outbreaks have been extensively described, they have primarily focused on SNFs. Less has been published on the occurrence of COVID-19 in ALFs (*6*). National characterization of COVID-19 in ALFs is challenging because these facilities do not have a federal COVID-19 reporting requirement, unlike CMS-certified SNFs. However, many state and territorial health departments collect and publicly report COVID-19 data across various types of LTCFs as part of their surveillance activities.

Starting April 30, 2020, health department websites were systematically searched for LTCF COVID-19 surveillance data at least weekly so that ALFs with one or more COVID-19 cases, and cases or deaths among residents and staff members could be counted. Data availability and presentation varied widely by state. Some reporting states aggregated surveillance data for all ALFs. Others provided COVID-19 case or death counts for individual LTCFs by name. For states providing LTCF names but not facility type, ALFs were identified by linking the facility name and available address information to general public listings of ALFs and similar residential care facilities* from state regulatory authorities. Some reporting states provided the number of affected facilities, number of cases, or number of deaths among ALF residents and staff members. Other states reported cases associated with active COVID-19 outbreaks, only representing cases or deaths occurring within a recent time frame, as indicated by the state. For these latter states, when possible, cumulative counts were approximated by using maximum active numbers from outbreaks among available reports, or by combining numbers from active and inactive outbreaks. Statewide COVID-19 case counts in the general population were obtained from USAFacts.^†^ The proportions of deaths among cases were calculated for the statewide general population, ALF residents, ALF staff members, and ALF residents and staff members, where possible. The overall number of U.S. ALFs was obtained using public listings from state regulatory authorities. SAS (version 9.4; SAS Institute) and Python (version 3.6.8; Python Software Foundation) were used for data analysis and to perform facility-level linkages.

As of October 15, 2020, 39 states had publicly available data reporting one or more COVID-19 cases in an ALF. The start of reporting varied by state, and when provided, ranged from February 27 to April 30, 2020. Among the 39 states, 38 reported the total number of ALFs in their state, 23 reported the number of cases among ALF residents, 22 reported the number of cases among ALF staff members, and 33 reported the number of cases among ALF residents and staff members. COVID-19–associated death data were available from 28 states for ALF residents and staff members combined, but available from only 20 states for ALF residents alone, and from nine states for ALF staff members alone.

A total of 33,167 licensed ALFs and similar residential care facilities from 50 states and the District of Columbia were identified through state government regulatory websites. Among the 39 states with available data, 6,440 (22%) of 28,623 ALFs had one or more COVID-19 cases as of October 15, 2020, ranging from 1.3% of ALFs in Iowa to 92.8% of ALFs in Connecticut (Table 1). Ten states (Connecticut, Georgia, Indiana, Kentucky, Massachusetts, Mississippi, New Jersey, North Dakota, Utah, and Washington) reported one or more cases in ≥50% of ALFs. Overall, 27,965 cases of COVID-19 were reported in ALF residents and 17,799 in ALF staff members (Table 1); 5,469 associated deaths were reported in residents and 46 in staff members (Table 2). ALF residents and staff members accounted for 4.1% and 0.1%, respectively, of COVID-19-associated deaths in the general population (Table 2). Among the states with available data, 21.4% of ALF residents and 0.6% of ALF staff members with COVID-19 died, compared with 2.5% of persons with COVID-19 who died in these states overall (Table 3).

**TABLE 1:** COVID-19 cases among residents and staff members in assisted living facilities (ALFs) and the general population — 39 states, October 15, 2020*

Location	ALFs^†,§,¶^		COVID-19 cases**			
	Total ALFs (38 states)	ALFs with ≥1 case (39 states)	General population (33 states)	ALF residents (23 states)	ALF staff members (22 states)	ALF residents and staff members (33 states)
	No.	No. (%)	No.	No. (%)	No. (%)	No. (%)
National^††^						
Total	28,623^§§^	6,440(22.5)	6,033,180	27,965 (0.6)	17,799 (0.4)	60,751 (1.0)
Median (IQR)	275 (211–640)	115 (65–216)	108,139 (70,520–175,922)	891 (324–1,939)	521 (227–1,106)	1,234 (526–2,774)
State						
Arizona	2,122	376 (17.7)	—^§§^	—^§§^	—^§§^	—^§§^
Arkansas	172	51 (29.7)	96,523	112 (0.1)	111 (0.1)	223 (0.2)
California^¶¶^	7,364	388 (5.3)	861,887	3,600 (0.4)	2,295 (0.3)	5,895 (0.7)
Colorado	656	80 (12.2)	80,776	891 (1.1)	615 (0.8)	1,506 (1.9)
Connecticut	111	103 (92.8)	59,748	1,100 (1.8)	134 (0.2)	1,234 (2.1)
Delaware	33	8 (24.2)	—^§§^	—^§§^	—^§§^	—^§§^
Florida	3,113	450 (14.5)	741,631	1,502 (0.2)	754 (0.1)	2,256 (0.3)
Georgia	252	213 (84.5)	336,227	1,098 (0.3)	958 (0.3)	2,056 (0.6)
Hawaii	18	6 (33)	5,349	1 (—)	1 (—)	2 (—)
Idaho	280	115 (41.1)	50,610	—^§§^	—^§§^	1,347 (2.7)
Illinois	663	254 (38.3)	331,613	—^§§^	—^§§^	3,977 (1.2)
Indiana	211	134 (63.5)	143,911	626 (0.4)	302 (0.2)	928 (0.6)
Iowa	455	6 (1.3)	103,222	—^§§^	—^§§^	106 (0.1)
Kansas	220	12 (5.5)	70,520	—^§§^	—^§§^	163 (0.2)
Kentucky	131	67 (51.1)	84,195	107 (0.1)	151 (0.2)	258 (0.3)
Louisiana	—***	185 (—***)	173,088	—^§§^	—^§§^	1,245 (0.7)
Maryland	1,626	201 (12.4)	133,547	2,253 (1.7)	1,952 (1.5)	4,205 (3.1)
Massachusetts	269	215 (79.9)	148,756	—^§§^	—^§§^	1,855 (1.2)
Minnesota	1,744	303 (17.4)	—^§§^	—^§§^	—^§§^	—^§§^
Mississippi	113	58 (51.3)	108,139	362 (0.3)	289 (0.3)	651 (0.6)
Montana	211	71 (33.6)	20,210	—^§§^	—^§§^	526 (2.6)
Nevada	381	94 (24.7)	87,968	481 (0.5)	274 (0.3)	755 (0.9)
New Hampshire	139	12 (8.6)	8,878	285 (3.2)	211 (2.4)	496 (5.6)
New Jersey	263	216 (82.1)	216,994	4,367 (2.0)	2,783 (1.3)	7,150 (3.3)
New Mexico	267	70 (26.2)	—^§§^	—^§§^	—^§§^	—^§§^
New York	337	65 (19.3)	—^§§^	—^§§^	—^§§^	—^§§^
North Carolina	591	174 (29.4)	240,105	2,274 (0.9)	976 (0.4)	3,250 (1.4)
North Dakota	139	70 (50.4)	28,947	117 (0.4)	90 (0.3)	207 (0.7)
Ohio	787	329 (41.8)	173,665	1,625 (0.9)	1,149 (0.7)	2,774(1.6)
Oklahoma	211	99 (46.9)	103,836	—^§§^	—^§§^	713 (0.7)
Oregon	241	65 (27.0)	38,522	—^§§^	—^§§^	1,003 (2.6)
Pennsylvania	1,203	448 (37.2)	175,922	2,456 (1.4)	1,264 (0.7)	3,720 (2.1)
Rhode Island	65	15 (23.1)	25,698	266 (1.0)	—^§§^	266 (1.0)
South Carolina	501	190 (37.9)	158,883	1,152 (0.7)	620 (0.4)	1,772 (1.1)
Tennessee	378	47 (12.4)	211,001	138 (0.1)	127 (0.1)	265 (0.1)
Texas	2,016	629 (31.2)	759,395	2,739 (0.4)	2,317 (0.3)	5,056 (0.7)
Utah	236	156 (66.1)	90,491	413 (0.5)	426(0.5)	839 (0.9)
Virginia	565	191 (33.8)	162,923	—^§§^	—^§§^	4,052 (2.5)
Washington	539	274 (50.8)	—^§§^	—^§§^	—^§§^	—^§§^

**Abbreviations:** COVID-19 = coronavirus disease 2019; IQR = interquartile range.

* Data were accessed on October 15, 2020. The most recent data available varied across states from September 24, to October 15, 2020.

^†^ The following states reported COVID-19 in individual long-term care facilities, but did not specify facility type: Arkansas, Delaware, Georgia, Idaho, Illinois, Indiana, Iowa, Kansas, Kentucky, Maryland, Minnesota, Mississippi, New Hampshire, New Jersey, New Mexico, New York, North Carolina, Oklahoma, and Oregon. For these states, facilities were linked with state regulatory listings to identify assisted living and similar residential care facilities.

^§^ “Assisted living facility” also refers to long-term care facilities defined as adult care facility (ACF), adult care home (ACH), adult home (AH), adult residential facility (ARF), assisted care living facility (ACLF), assisted care living home (ACLH), assisted living community (ALC), assisted living facility special care (ALF SC), assisted living program (ALP), assisted living residence (ALR), basic care facility (BCF), community residential care facility (CRCF), enriched housing program (EHP), home for the aged (HFTA), personal care home (PCH), residential care facility (RCF), residential care facility for the elderly (RCFE), supportive living program (SLP), and supported residential care facility (sRCF). The following states report COVID-19 in assisted living facilities using one or more of those terms: California (ARF, RCFE), Illinois (ALF, SLP), Indiana (RCF), Iowa (ALF, RCF), Louisiana (ARF), Maryland, (ALP), Massachusetts (ALR), New Hampshire (sRCF, ALF, RCF), New Jersey (ALR, ALP, PCH), New York (AH, ALP, EHP), North Carolina (ACH), North Dakota (ALF, BCF), Oklahoma (ALC, RCF), Pennsylvania (ALF, ALF-SC, PCH), South Carolina (CRCF), and Tennessee (ACLF, HFTA).

^¶^ Numbers reflect cumulative counts where available. The following states report cases associated with active COVID-19 outbreaks, which represent those occurring in a recent timeframe, as defined by the state: Delaware, Florida, Mississippi, New Jersey, North Carolina, North Dakota, Tennessee, and Utah. When possible, cumulative counts were approximated by using maximum active numbers from outbreaks among available reports retrieved over time (Delaware, Mississippi, New Jersey, North Carolina, and North Dakota) or by combining numbers from active and inactive outbreaks (Colorado).

** ALF COVID-19 case data come from state health department websites; general population COVID-19 case data come from https://usafacts.org/visualizations/coronavirus-covid-19-spread-map/.

^††^ Each state-level measure was summed to the national level and summarized across reporting states using median and IQR.

^§§^ No data presented.

^¶¶^ When ranges were used instead of actual numbers, the minimum non-zero number within the range was used (e.g., a value of “<5” was treated as a count of 1); applicable states included: California and Massachusetts.

*** A reliable estimate was not ascertainable for the total number of facilities corresponding to those reported on by the state health department for Louisiana and therefore does not contribute to the total number of ALFs.

**TABLE 2:** COVID-19-associated deaths*^,^^†^ among residents and staff members in assisted living facilities (ALFs) and the general population — 28 states, October 15, 2020^§,¶^

Location	General population (28 states) No.	ALF residents (20 states) No. (%)	ALF staff members (9 states) No. (%)	ALF residents and staff members (28 states) No. (%)
Nationwide**				
Total	153,348	5,469 (4.1)	46 (0.1)	7,433 (4.8)
Median (IQR)	3,269 (1,136–5,993)	215 (61–397)	1 (0–5)	156 (67–403)
State				
Arkansas	1,636	15 (0.9)	0 (—)	15 (0.9)
California	16,677	444 (2.7)	24 (0.1)	468 (2.8)
Colorado	2,159	229 (10.6)	0 (—)	229 (10.6)
Connecticut	4,527	381 (8.4)	0 (—)	381 (8.4)
Delaware	651	37 (5.7)	—^††^	37 (5.7)
Georgia	7,486	230 (3.1)	—^††^	230 (3.1)
Idaho	517	—^††^	—^††^	151 (29.2)
Illinois	9,127	—^††^	—^††^	692 (7.6)
Indiana	3,862	160 (4.1)	—^††^	160 (4.1)
Kentucky	1,296	18 (1.4)	0 (—)	18 (1.4)
Louisiana	5,495	—^††^	—^††^	146 (2.7)
Maryland	4,086	587 (14.4)	12 (0.3)	599 (14.7)
Mississippi	3,152	72 (2.3)	—^††^	72 (2.3)
Montana	227	—^††^	—^††^	38 (16.7)
Nevada	1,691	98 (5.8)	1 (0.1)	99 (5.9)
New Hampshire	448	—^††^	—^††^	82 (18.3)
New Jersey	16,197	1,326 (8.2)	—^††^	1,354 (8.4)
New York	33,041	200 (0.6)	—^††^	200 (0.6)
North Carolina	3,874	303 (7.8)	5 (0.1)	308 (8.0)
Oklahoma	1,143	—^††^	—^††^	65 (5.7)
Oregon	610	—^††^	—^††^	121 (19.8)
Pennsylvania	8,410	483 (5.7)	—^††^	483 (5.7)
Rhode Island	1,116	42 (3.8)	—^††^	42 (3.8)
South Carolina	3,575	253 (7.1)	4 (0.1)	257 (7.2)
Tennessee	2,726	20 (0.7)	—^††^	20 (0.7)
Texas	15,702	504 (3.2)	—^††^	504 (3.2)
Utah	531	67 (12.6)	—^††^	67 (12.6)
Virginia	3,386	—^††^	—^††^	595 (17.6)

**Abbreviations:** COVID-19 = coronavirus disease 2019; IQR = interquartile range.

* The following states reported COVID-19 deaths in individual long-term care facilities, but did not specify facility type: Arkansas, Delaware, Georgia, Idaho, Illinois, Indiana, Iowa, Kansas, Kentucky, Maryland, Minnesota, Mississippi, New Hampshire, New Jersey, New Mexico, New York, North Carolina, Oklahoma, and Oregon. For these states, facilities were linked with state regulatory listings to identify assisted living and similar residential care facilities.

^†^ Numbers reflect cumulative counts where available. The following states only report COVID-19–related deaths in active outbreaks, which represent those occurring in a recent timeframe, as defined by the state: Delaware, Mississippi, New Jersey, North Carolina, Tennessee, and Utah. When possible, cumulative counts were approximated by using maximum active numbers from outbreaks among available reports retrieved over time (Delaware, Mississippi, New Jersey, North Carolina, and North Dakota).

^§^ Data were accessed on October 15, 2020. The most recent data available varied across states from September 24, to October 15, 2020.

^¶^ ALF COVID-19-associated death data come from state health department websites; general population COVID-19-associated death data come from https://usafacts.org/visualizations/coronavirus-covid-19-spread-map/.

** Each state-level measure was summed to the national level and summarized across reporting states using median and IQR.

^††^ No data presented.

**TABLE 3:** Proportion of deaths among COVID-19 cases*^,^^†^ among residents and staff members in assisted living facilities (ALFs) and general population — 26 states, October 15, 2020^§^

Location	General population (25 states)	ALF residents (17 states)	ALF staff members (9 states)	ALF residents and staff members (25 states)
	% (No. deaths/cases)	% (No. deaths/cases)	% (No. deaths/cases)	% (No. deaths/cases)
Nationwide^¶^				
Total	2.5 (119,656/4,761,090)	21.4 (5,232/24,435)	0.6 (46/7,128)	13.5 (7,196/53,388)
Median (IQR)	2.2 (1.6–3.0)	19.8 (15.9–24.7)	0.4 (0–0.6)	11.9 (9.2–15.1)
State				
Arkansas	1.7 (1,636/96,523)	13.4 (15/112)	0.0 (0/111)	6.7 (15/223)
California	1.9 (16,677/861,887)	12.3 (444/3,600)	1.0 (24/2,295)	7.9 (468/5,895)
Colorado	2.7 (2,159/80,776)	25.7 (229/891)	0.0 (0/615)	15.2 (229/1,506)
Connecticut	7.6 (4,527/59,748)	34.6 (381/1,100)	0.0 (0/134)	30.9 (381/1,234)
Georgia	2.2 (7,486/336,227)	20.9(230/1,098)	—**	11.2 (230/2,056)
Idaho	1.0 (517/50,610)	—**	—**	11.2 (151/1,347)
Illinois	2.8 (9,127/331,613)	—**	—**	17.4 (692/3,977)
Indiana	2.7 (3,862/143,911)	25.6 (160/626)	—**	17.2 (160/928)
Kentucky	1.5 (1,296/84,195)	16.8 (18/107)	0.0 (0/151)	7.0 (18/258)
Louisiana	3.2 (5,495/173,088)	—**	—**	11.7 (146/1,245)
Maryland	3.1 (4,086/133,547)	26.1 (587/2,253)	0.6 (12/1,952)	14.2 (599/4,205)
Mississippi	2.9 (3,152/108,139)	19.9 (72/362)	—**	11.1 (72/651)
Montana	1.1 (227/20,210)	—**	—**	7.2 (38/526)
Nevada	1.9 (1,691/87,968)	20.4 (98/481)	0.4 (1/274)	13.1 (99/755)
New Hampshire	5.0 (448/8,878)	—**	—**	16.5 (82/496)
New Jersey	7.5 (16,197/216,994)	30.4 (1,326/4,367)		18.9 (1,354/7,150)
North Carolina	1.6 (3,874/240,105)	13.3 (303/2,274)	0.5(5/976)	9.5 (308/3,250)
Oklahoma	1.1 (1,143/103,836)	—**	—**	9.1 (65/713)
Oregon	1.6 (610/38,522)	—**	—**	12.1 (121/1,003)
Pennsylvania	4.8 (8,410/175,922)	19.7 (483/2,456)	—**	13.0 (483/3,720)
Rhode Island	4.3 (1,116/25,698)	15.8 (42/266)	—**	15.8 (42/266)
South Carolina	2.3 (3,575/158,883)	22.0 (253/1,152)	0.6 (4/620)	14.5 (257/1,772)
Tennessee	1.3 (2,726/211,001)	14.5 (20/138)	—**	7.5 (20/265)
Texas	2.1 (15,702/759,395)	18.4 (504/2,739)	—**	10.0 (504/5,056)
Utah	0.6 (531/90,491)	16.1 (67/413)	—**	8.0 (67/839)
Virginia	2.1 (3,386/162,923)	—**	—**	14.7(595/4,052)

**Abbreviations:** COVID-19 = coronavirus disease 2019; IQR = interquartile range.

* ALF COVID-19–associated case and death data come from state health department websites; general population COVID-19–associated case and death data come from https://usafacts.org/visualizations/coronavirus-covid-19-spread-map/.

^†^ Proportion of deaths among COVID-19 cases was calculated by dividing deaths by cases.

^§^ Data were accessed on October 15, 2020. The most recent data available varied across states from September 24, to October 15, 2020.

^¶^ Each state-level measure was summed to the national level and summarized across reporting states using median and IQR.

** No data presented.

## Discussion

As of October 15, 2020, an average of one death occurred among every five ALF residents with COVID-19, compared with one death among every 40 persons in the general population with COVID-19 in states with available data. Wide variability was observed across states in the proportion of ALFs with one or more residents and staff members with COVID-19, ranging from 1% to 93%. Statewide COVID-19 incidence and reporting practices might in part explain this variability. Such findings indicate the need to continue monitoring the effect of COVID-19 in ALFs and for infection prevention and control recommendations to be recognized and followed (*2*).

SARS-CoV-2 transmission can occur within LTCFs, among and between residents and staff members. ALFs are at risk for several reasons, including the congregate nature of the setting and need for close contact between staff members and residents as part of care (*7*). Community-acquired infections among staff members can also contribute to the introduction of SARS-CoV-2 into LTCFs (*8*). On average, residents are at increased risk for severe COVID-19–related outcomes because of their age and higher prevalence of chronic conditions (*9*). As of August 6, 2020, a similar resident proportion of deaths among COVID-19 patients (22%) was observed in nine states reporting cumulative numbers of cases and deaths among ALFs and similar residential care facilities (*10*).

The findings in this report are subject to at least five limitations. First, because data on COVID-19 in ALFs from 11 states, the District of Columbia, and six territories could not be ascertained, the findings in this report might not be representative of all U.S. ALFs, residents, and staff members. Second, for the states reporting facility-level LTCF counts, linkage to names and address information from regulatory records was required to identify ALFs; those records might have been incomplete or the process might have misclassified facilities. Third, comparisons between states were limited by variation in publicly reported count types on health department websites (e.g., cumulative versus active), level of aggregation (e.g., state, county, or facility level), population (e.g., residents, staff members, or both), and a lack of standardization in ALF definitions. Fourth, delays in testing residents and staff members early in the pandemic, differences in when states began requiring and publicly posting LTCF data, and changes in surveillance methods during the pandemic might have resulted in underestimations of the numbers of affected facilities, cases, and deaths among ALF residents and staff members. Finally, with only a small number of states publicly reporting deaths among ALF staff members, these data should be interpreted with caution and might not be generalizable to the national level.

State and territorial health department websites are important sources of publicly available COVID-19 surveillance data from ALFs. National surveillance data are less readily available for ALFs. Increased standardization in public reporting format across states could improve the characterization of COVID-19 in these LTCFs across the United States. Although ALFs do not have the same federal reporting requirements as do CMS-certified SNFs, ALFs can voluntarily report COVID-19 cases, facility staffing, and supply information to the CDC National Healthcare Safety Network LTCF COVID-19 module.^§^ Innovative uses of COVID-19 surveillance data from ALFs can focus resources and inform prevention and response activities and might have implications for vaccine programs. The disproportionate share of deaths among ALF residents underscores the need for ongoing surveillance of nationwide COVID-19 data and more robust infection prevention and control activities to protect this population.

ALFs, like all LTCFs, should remain vigilant to prevent the introduction and spread of SARS-CoV-2 in their facilities. Preventive steps should include 1) identifying a point of contact at the local health department to aid prompt notification; 2) educating residents, family members, and staff members about COVID-19; 3) having a plan for visitor and staff member restrictions; 4) encouraging social (physical) distancing and the use of masks, as appropriate; 5) implementing recommended infection prevention and control practices and providing access to supplies; 6) rapidly identifying and properly responding to residents and staff members with suspected or confirmed COVID-19; and 7) conducting surveillance of COVID-19 cases and deaths, facility staffing, and supply information (*2*).

**Summary**

**What is already known about this topic?**

Although the spread of SARS-CoV-2 in nursing homes is well documented, relatively little has been reported on COVID-19 among residents and staff members in U.S. assisted living facilities (ALFs).

**What is added by this report?**

By October 15, 2020, in 39 states with available data, 22% of ALFs reported one or more cases of COVID-19 among residents and staff members. Among ALF residents with COVID-19, 21% died, compared with 3% who died among the general population with COVID-19.

**What are the implications for public health practice?**

With ongoing community transmission, ALFs should take actions to prevent the spread of SARS-CoV-2 in their facilities, including rapid identification and response to residents and staff members with suspected or confirmed COVID-19.

## Footnotes

* “Assisted living facility” also refers to long-term care facilities defined as adult care facility (ACF), adult care home (ACH), adult home (AH), adult residential facility (ARF), assisted care living facility (ACLF), assisted care living home (ACLH), assisted living community (ALC), assisted living facility special care (ALF SC), assisted living program (ALP), assisted living residence (ALR), community residential care facility (CRCF), home for the aged (HFTA), personal care home (PCH), residential care facility (RCF), residential care facility for the elderly (RCFE), supportive living program (SLP), and supported residential care facility (sRCF). The following states report COVID-19 in assisted living facilities using one or more of those terms: California (ARF, RCFE), Illinois (ALF, SLP), Indiana (RCF), Iowa (ALF, RCF), Louisiana (ARF), Maryland, (ALP), Massachusetts (ALR), New Hampshire (sRCF, ALF, RCF), New Jersey (ALR, ALP, PCH), New York (AH, ALP, EHP), North Carolina (ACH), Oklahoma (ALC), Pennsylvania (ALF, ALF-SC, PCH), South Carolina (CRCF), and Tennessee (ACLF, HFTA).

† https://usafacts.org/.

§ https://www.cdc.gov/nhsn/ltc/covid19/index.html.
